# Fabrication and Characterization of a Flexible Polyurethane-Based Triboelectric Nanogenerator for a Harvesting Energy System

**DOI:** 10.3390/mi16020230

**Published:** 2025-02-17

**Authors:** Saba Ejaz, Imran Shah, Shahid Aziz, Gul Hassan, Ahmed Shuja, Muhammad Asif Khan, Dong-Won Jung

**Affiliations:** 1 Department of Electrical and Computer Engineering, International Islamic University, Islamabad 44000, Pakistan; saba.ejaz028@gmail.com; 2 Centre for Advanced Electronics and Photovoltaic Engineering (CAEPE), International Islamic University, Islamabad 44000, Pakistan; ahmed.shuja@iiu.edu.pk; 3 Department of Mechatronics Engineering, Air University, Islamabad 44000, Pakistan; imranshahswabi@gmail.com; 4 Department of Mechanical Engineering, Jeju National University, 102 Jejudaehak-ro, Jeju-si 63243, Republic of Korea; shahid@jejunu.ac.kr; 5 Department of Mechanical Engineering, International Islamic University, Islamabad 44000, Pakistan; asif.khan@iiu.edu.pk

**Keywords:** triboelectric nanogenerator (TENG), polyurethane (PU), aluminum electrode (Al)

## Abstract

Powering wearable and portable devices, triboelectric nanogenerators (TENGs) are a considerably promising technology. Low-cost production, ease of fabrication, optimal efficiency, and high output performance are always key concerns in developing energy harvesting technologies. Optimum efficiency and high output are always key concerns. This research addresses the ongoing challenge of raising efficient, flexible, and lightweight energy harvesting systems for recent wearable technologies. In this research, a triboelectric nanogenerator is proposed for harvesting the triboelectric effect. Using polyurethane (PU), a bendable TENG that is in the vertical contact separation mode was developed. UV-curable PU forms the basis of TENGs. A sponge, repurposed from landfill waste, acts by means of a spacer to maintain a consistent air gap between the tribo-layers for enhanced triboelectrification. The triboelectric nanogenerators formed a V_oc_ approaching 500 V and a current of ~2 µA and also showed high performance with a power density of 8.53 W/m^2^. In addition, the triboelectric nanogenerator can light LEDs and charge capacitors, making it a self-powered energy source for portable devices, Wi-Fi, and monitoring systems. The proposed TENG provides a capable solution for sustainable, self-powered wearable electronics and has the potential for further development in energy-efficient and eco-friendly applications.

## 1. Introduction

As sustainable global development gains popularity, scientists are becoming more conscious of green energy and materials [1]. Human biomechanical energy continually affects its surroundings. If converted into electricity, it has huge promise as an energy source for wearable sensors. Therefore, we must efficiently gather biomechanical energy [2]. Triboelectric mechanisms outperform piezoelectric and electromagnetic mechanisms. Triboelectric nanogenerators (TENGs) have garnered widespread scientific interest since their creation [3]. Minmin, Wang, et al. used triboelectrification and electrostatic induction to convert mechanical energy from the environment into electrical energy [4]. Yang, Jiang et al. revealed that it has self-powered sensing, which is common in multifunctional sensors due to its affordability, variety in material choices, and affordability [5]. V. Palaniappan et al. proposed four TENG modes: contact separation, single electrode, free-standing, and cross-sliding. In any of these modes, when electrodes are automatically activated, potential differences and charge transmission occur [6]. Wei-Chen, Gao, et al. stated that polyurethane has received attention in smart material research due to its excellent mechanical properties, high compatibility, and simplicity of modification [7]. Danielli et al. said that reversible dynamic covalent bonds are one of the best ways to make things self-healing because they can get around problems with recycling and processing thermosetting polymers [8]. Noon et al. presented that current research focuses on aspects that improve biogas generation. Significant factors include codigestion, substrate size, temperature, pH, and catalyst addition. Three batch operations took 21 days in mesophilic conditions, making them easier to achieve than thermophilic ones. Combining cow manure, food, poultry waste, and sewerage water demonstrated promising outcomes compared to cow dung alone [9].

It was found by Zhong, Lin, et al. that supercapacitors and batteries can self-charge by combining energy harvesting devices that use pyroelectric, thermoelectric, photovoltaic, piezoelectric, and triboelectric processes [10]. Anwar et al. proposed that the current study evaluates wind and solar energy and chooses the best energy systems with minimum levelized energy systems for Jeju Island, South Korea’s largest island, using Home Pro software (Version 3.18.1). This study’s initial wind conditions are explained using Weibull and mean wind speed graphs. We assess the wind potential of each region using size and form parameters. Southwest winds were more common than southeast winds [11].

Zhang et al. proposed that they created a flexible triboelectric nanogenerator (TENG) for energy gathering. The TENG consists of a graphite/polydimethylsiloxane (G/PDMS) composite on a copper (Cu) anode and a PVDF layer on an aluminum (Al) cathode [12]. Huang et al. described that they created a book-shaped triboelectric nanogenerator (TENG) using electrospun PVDF and PHBV nanofibers to gather mechanical energy. Graphene oxide that is spread out in PVDF nanofibers traps charges, which makes the TENG’s charge storage interface and output better [13]. Yan Shao et al. presented an innovative and practical method for making an all-in-one triboelectric nanogenerator (TENG). The TENG was made from rubber/carbon nanofiber composites using supercritical CO_2_ foaming, which is eco-friendly and mass-produced. When everything was perfect, the structure obtained an I_SC_ value of 2.87 μA, a V_OC_ of 91 V, and a conveyed charge of 40 nC. This was 10 times more than the single-layer TENG in the same 4 cm² area [14]. Shaukat et al. presented a review covering advances in TENG device recycling and biowaste material. The potential of natural and oceanic biowaste is also considered. Lastly, we provide prospects for the structural development of TENG devices [15]. Gul Hassan et al. proposed a hybrid NG to gather piezoelectric and triboelectric effects simultaneously. The suggested hybrid NG uses polydimethylsiloxane (PDMS) and perovskite zinc stannite (ZnSnO_3_) nanocubes to create piezoelectric and triboelectric NGs with a high charge polarization of 59 µC cm^−2^ for composite (PDMS + ZnSnO_3_) and UV surface-treated PDMS, respectively. To obtain both the high output current of PENGs and the high voltage of TENGs, these two NGs are stacked on top of each other and spaced apart with sponge spacers. This creates an even air gap for the triboelectric effect [16]. Iftikhar Ali et al. presented that as portable electronics and self-powered systems become more popular, researchers are creating nanogenerators as energy sources. High productivity and efficiency are always priorities. In this research, we devise and use a hybrid NG, based on piezoelectric and triboelectric phenomena, to gather wind energy. The TENG and PENG are made using UV-curable polyurethane (PU) and a powdered zinc oxide (ZnO) composite (ZnO + PU) [17].

Hyun, J. Oh, et al. made a triboelectric nanogenerator (TENG) that can heal itself using a linear silicone-modified polyurethane (PU) covering. The PU covering was created by mixing hydroxypropyl silicone oil and hexamethylene diisocyanate with the help of Sn [18]. A triboelectric nanogenerator (TENG) was created in 2012 to capture mechanical energy from ambient sources to feed scattered energy sources, according to Jing, Yan, et al. The TENG is based on Maxwell’s equations [19]. According to Weixiang, Sun et al., a stretchy TENG made of liquid metal, hydrogen, and water exhibits a stretchability of over 2500% [20]. Yike, Liu et al. stated that triboelectric nanogenerators (TENGs) have contributed to ocean energy storage development over the past decade, bringing many benefits. Wind farms use electromagnetic technology, and their turbine noise is an ecological issue [21]. Masahiro, Matsunaga, et al. reported a rubber-constructed stretchable triboelectric device with a signal electrode that transmitted control between the ground and electrode due to in-plane charge dispersion. One can use this approach to identify motion direction. We also presented stretchy single-electrode TENGs with conductive fabric bases [22]. Wei Wang, Jin Yan, and others created liquid–solid triboelectric nanogenerators (L-S TENGs) for collecting liquid energy and fast self-powered sensing. This research uses a “Multiphase Liquid Triboelectric Nanogenerator Prototype (ML-TENG Pro)” that is made up of lubricating oil and deionized water as dielectric materials. It is based on a solid–liquid nanogenerator with a single electrode [23]. Huaifang, Qin, et al. found auxetic polyurethane (APU)-TENG in the horizontal strip of the safety belt. This allows you to keep an eye on the driver’s forward position from afar, even when the vehicle is slowing down quickly and violently. It does this with a high level of sensitivity (0.89 V/cm^2^) for measuring strain in a wide range of ranges (40% to 100%) [24]. Fang, Yi, et al. created self-healing triboelectric nanogenerators (SH-TENGs) by combining the triboelectric effect with electrostatic induction and the ability to heal itself. They demonstrate the use of a self-healing and mechanically resilient poly(hindered urea) (PHU) network to create efficient TENGs [25]. Yuanxiao, Feng, et al. claim that a sponge-based TENG with conductive MWCNTs and a PU sponge can elicit an open-circuit voltage of 110 V and a short-circuit current of 12 μA [26]. Aso Ali Abdalmohammed Shateri et al. introduced the double horizontal linear-to-rotational triboelectric nanogenerator (DHLR-TENG). This novel method converts horizontal linear mechanical energy into electrical energy using a gear arrangement. The testing results showed that the DHLR-TENG produces a full AC cycle when integrated into an electrical circuit. It regularly performs well with an open-circuit voltage of 544 V, a short-circuit current of 61.16 µA, and a maximum power output of 33.27 mW [27]. PDMS networks with dynamic imaging bonds cure 94% of mechanical flaws, according to Patel et al. On the other hand, they use mechanical elastomer recovery to electrically cure the buckled electrode [28].

Hong-Zhi, Ma et al. suggested using shape memory polymers (SMPs) to make TENGs last longer and work better. This paper describes a new smart SMP-based self-healing TENG that can fix itself and return to full performance after the triboelectric layer breaks down. The SMP-TENG’s deterioration, mending, and increased lifetime and endurance show its outstanding potential [29]. Jiangman, Sun, et al. reported that deformable or wearable energy producers with high stretch and mending abilities are still difficult to construct. To get around this problem, they made a thermoplastic elastomer composite with liquid metal and silver flakes that can be used as stretchy conductors for TENGs. This compound is stretchy, healable, and conductive. The elastomer is the conductor’s matrix and triboelectric layer. The nanogenerator’s super molecular hydrogen bonding with the thermoplastic elastomer fixed its energy-gathering ability after being severely damaged by mechanical forces and made it 250% more stretchable [30].

Jeong, Hwan et al. found that the inability to properly clean wearable electronics and an external power source are the two biggest challenges that need immediate attention. They demonstrated the full potential of TENGs by using a self-powered e-wristband as a keyboard to operate basic computer activities. The TENGs produced a power output of 1.25 W/m^2^ with an open-circuit voltage of −162 V and a short-circuit current of −42 µA [31]. Pengcheng Zhu, Shuairong Mu, and their colleagues created a soft and multifunctional neurological E-skin (SMNE) that has a poly(3-hexylthiophene) (P3HT) nanofiber polymer semiconductor-based stretchable synaptic transistor and several soft artificial sensory receptors that can pick up on light, heat, and force. The stretchable synaptic transistor converts electrical impulses into transient channel currents such as biological excitatory postsynaptic currents [32]. Kaushik, Parida, et al. discovered that putting together polyurethane and PTFE layers produces the best TENG properties. When gently tapped with 0.33 N, the output voltage reached 33.5 V and the current was 27.4 μA. Image analysis and atomic force microscopy detailed the topography of the film surface. Surface roughness was strongly correlated with the TENG’s electrical output, albeit not linearly [33]. An ultra-stretchable TENG (US-TENG) can cure an abrasion and a fracture at room temperature, according to Doga, Doganay, et al. Polydimethylsiloxane chains with hydrogen bonds and dynamic metal–ligand coordination can create synthetic elastomers with 10,000% stretchability and 100% self-healing at room temperature [34]. In their research, D. Satturappa, R. Srither, and others talked about how conjugated microporous polymers (CMPs) can be used as triboelectric materials for triboelectric nanogenerators (TENGs) and how much energy they can store. The Sonogashira technique combined 1,3,5-triethynylbenzene and 1,4-diiodoarenes to form nano- or submicron-sized CMP particles (CMP-X). Optimization of CMP-X nanoparticles in a polyurethane matrix resulted in top-notch triboelectrification performance, achieving a maximum power density of 0.80 mW cm^−2^ and p-p voltages of 411 V [35]. Jinxing, Jiang, Q. et al. created a self-contained electrospinning system with an R-TENG, VDRC, and spinneret. The R-TENG generates 1400 volts of alternating electricity. A voltage-doubling rectifying circuit may achieve a continuous direct voltage of 8.0 kV under ideal conditions. Electrospinning can be performed with this voltage to make nanofibers out of thermoplastic polyurethanes, polyamide-6 (PA6), polyacrylonitrile (PAN), polyethylene terephthalate (PET), and polyvinylidene difluoride (PVDF) [36].

R.D.I.G. Dharmasena et al. created a method that uses the same triboelectric materials as TENG contact surfaces and obtained results similar to those of triboelectrically different materials, which increases the range of materials that can be used in TENGs. They also talked about how materials move across TENG contact surfaces and how that affects nonlinear charging behavior from the outside. This showed a lot of important factors that impact how reliable and effective TENGs are [37]. In Shujie Yang et al.’s self-powered sport sensor system, graphene and polytetrafluoroethylene (PTFE) friction materials are mixed with a thermoplastic polyurethane (TPU) film. GT-TENG is a graphene-doped TPU nanocomposite film-based TENG with excellent durability [38]. Jose Miguel Blancas Flores et al. suggest that to obtain the best TENG design, we should think about the mechanical parameters, the nanofiller content, and the surface physicochemical properties of materials that can be stretched and which have better mechanical properties [39].

**Figure 1:**
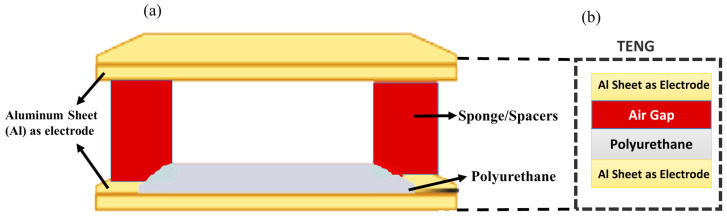
(**a**) Structure of the proposed TENG. (**b**) Zoomed-in structure of the TENG.

This research designs a polyurethane (PU) triboelectric nanogenerator for energy system harvesting. The PU-TENG is tested under varied electrical loads and charging storage devices to demonstrate mechanical energy harvesting. Figure 1 presents the proposed TENG structure. The proposed PU-TENG performs well in recognizing and harvesting the energy from applications. The developed TENG not only opens ways to repurpose these aluminum foil and landfill sponge materials but also shows good performance, with a power density of 8.53 W/m^2^. This is enough energy to power a light consisting of 360 LEDs while charging a 10 µF capacitor of up to 3 V in less than a minute. The developed TENG can be installed in shoes and harvest human motion while functioning as a self-powered motion detector and step counter.

## 2. Methodology and Experimentation

The four basic modes of the TENG are the vertical contact separation mode, in-plane contact sliding mode, single electrode (SE) mode, and freestanding triboelectric layer (FTL) mode. In our research, the vertical contact separation mode for TENGs is used.

### 2.1. Vertical Contact Separation Mode

**Figure 2:**
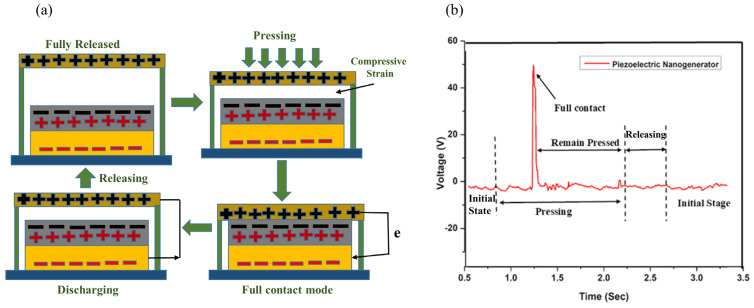
(**a**) Complete mechanism process of the proposed TENG. (**b**) Generated Output voltage.

The constructed triboelectric NG device’s operational processes and output response are detailed in Figure 2a. Figure 2b shows the TENG’s output voltages, clarifying Figure 2a’s method. As seen in Figure 2a, the gadget starts in steady conditions without external effort. Figure 2b exhibits noise despite inaccurate voltage generation due to environmental variables and light. Externally pressing the device with hand-tapping force helps explain the mechanism. Step 2 begins the device’s finger tapping and pressing phase (Figure 2a). Charges build and the output curve changes, as shown in Figure 2b. The TENG’s peak voltage reached 80 volts in step 3 of Figure 2a. The device’s PU layer and Al electrode are entirely contracted. The gadget releases pressure and stabilizes after contracting, just like before. As shown in Figure 2b, discharging events during triboelectrification caused a negative peak upon release. This changes the electric field in Figure 2a and reverses triboelectric charges in the external circuit. The positive and negative peak values are not the same because the device’s external force and the layers’ or substrates’ restorative forces do not match.

### 2.2. Design Principle

**Figure 3:**
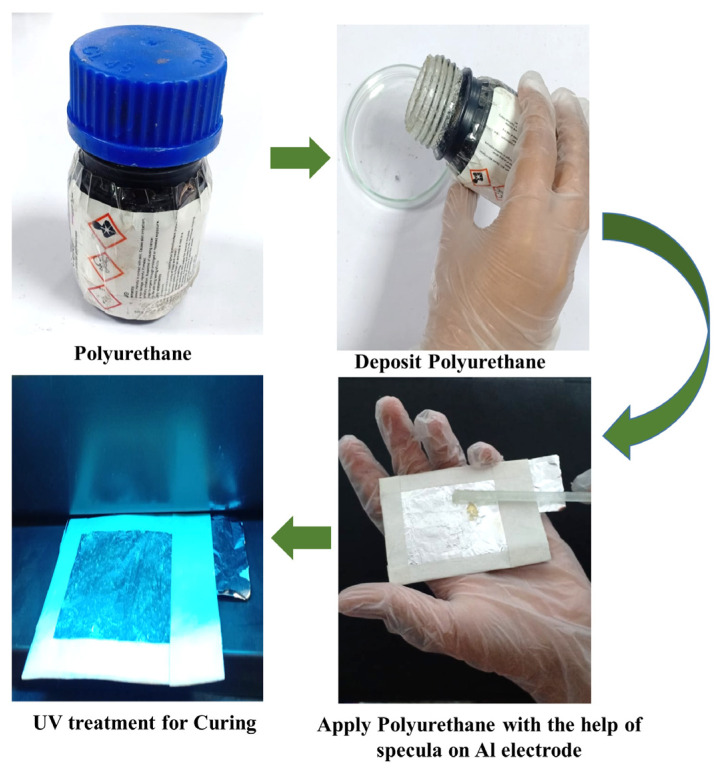
Design principle of the TENG.

In the design principle of the triboelectric nanogenerator, first, a PET sheet is taken and covered with aluminum foil, which acts as an electrode. Then, polyurethane (PU) is taken into the darkroom to melt as a thin solution because polyurethane (PU) is a versatile synthetic polymer used to make triboelectric nanogenerators (TENGs) to generate electricity. Polyurethane is applied in the middle of the bottom electrode layer-by-layer with the help of a spatula to create a very thin smooth layer and act as a UV treatment for curing the PU layers smoothly. The UV treatment is provided to cure the surface and make it smooth, as shown in Figure 3. Once it is completely dry, another top electrode is made with a PET sheet covered with aluminum foil.

### 2.3. Fabrication Processes

**Figure 4:**
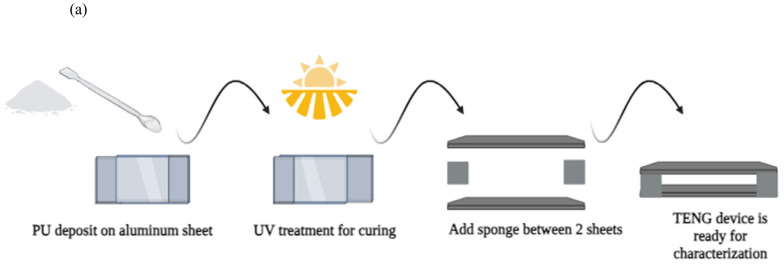

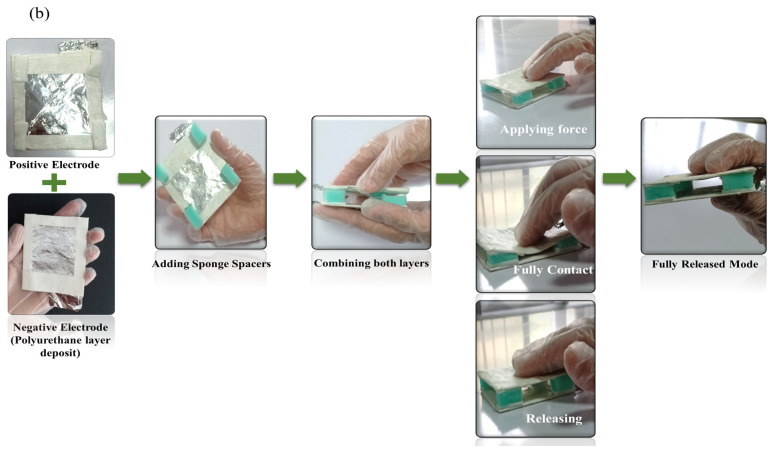
(**a**) Schematic Design. (**b**) Fabrication Process of the TENG.

Figure 4a presents an overview of the manufacturing process of the TENG device. UV-curable polyurethane (PU) polymer was acquired from Photopolymer Pts Ltd. (Singapore) and utilized in the device’s fabrication. Ultraviolet wavelengths are considered very effective electromagnetic wavelengths due to their ability to rapidly cure, dry, and solidify many materials, including polymer resins, adhesive films, dyes, and paints. We used a UV nail lamp (Model LN-818P, Ningbo Joystar tools Co., Ltd., Ningbo, China) for UV curing, which reduces adhesion and hardens and dries the applied layer. We subjected the fabricated devices to the UV ozone for ten minutes within a sealed, transparent glass cylindrical container. Figure 4a also displays an image of the synthesized TENG layer on aluminum foil.

The planned triboelectric NG device undergoes fabrication. TENG gadgets only employ polyurethane. A sponge separates the TENG, ensuring triboelectric phenomena between the Al electrode and the PU layer. The suggested TENG combines two Al electrodes—one on top and one on the bottom—with PU on that layer. Cost-effective, flexible, and high-performance TENGs are developed for harvesting ambient mechanical energy, such as human motion. In the developed TENG device, landfill and recyclable materials are repurposed, with ultraviolet (UV) light-curable polyurethane (PU) and Kapton tape as a tribo-layer. A sponge, repurposed from landfill waste, acts as a spacer to maintain a consistent air gap between the tribo-layers for enhanced triboelectrification, while recycled aluminum kitchen foil is utilized as electrodes. The developed TENG not only opens ways to repurpose these aluminum foil and landfill sponge materials but also shows strong performance, with a power density of 8.53 W/m^2^. The coated layer was cured under UV light for 30 min to ensure complete drying. Two electrodes are layered and separated by sponge spacers to create a homogeneous air gap for the triboelectric effect. The proposed design also uses sponge spacers for TENG contact separation, improving TENG performance. This arrangement harvests the generator’s vertical tension throughout the pushup/pressure cycle, as shown in Figure 4b.

## 3. Results and Discussion

This section presents the open-circuit voltage and short-circuit current results. The morphology of PU is discussed in this section.

**Figure 5:**
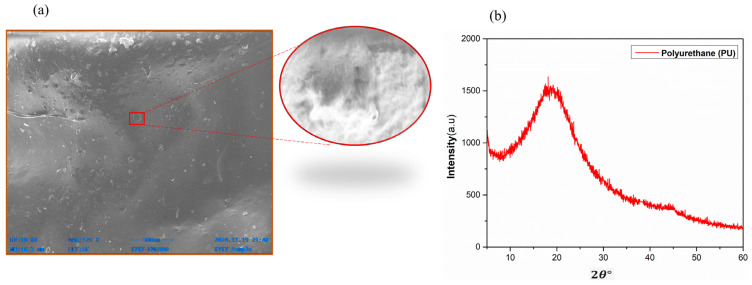
(**a**) SEM image of PU. (**b**) X-ray Diffraction (XRD) Results.

The domain morphology of PU can be seen via dark-field electron microscopy. With time, the soft segment domains in both as-cast and drawn films grow larger and more perfect. In Figure 5a, an SEM image of PU is shown. This image was extracted using SEM characterization. The width of the PU material lies in the range of 18.3 mm to 18.8 mm. The average diameter of PU is confirmed at 10 µm. For application in TENGs, PU provides a larger surface area to the contact layers, which contributes to an effective triboelectrification process. The X-ray diffraction (XRD) of PU is shown in Figure 5b. A wide diffraction peak can be seen at an angle of about 20°, which is close to the observations for other polyurethane materials. Polyurethane is a durable substance that resists tearing and cuts well. It is frequently utilized in industrial domains such as aviation, automobiles, and eyeglass frames.

**Figure 6:**
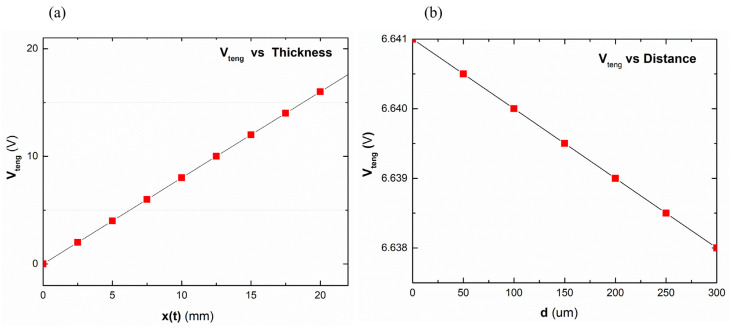
(**a**) Variation of the V_teng_ by the varied x(t). (**b**) Variation of the V_teng_ by the varied d.

Figure 6 illustrates how a few important geometric characteristics affect the device’s performance. Figure 6a shows the V_teng_ fluctuation with d and x(t). This figure provides potential TENG geometry synthesis settings, which are important to device performance. Theoretical analysis of contact mode TENG performance indicates that varying the triboelectric surface thickness, air gap, and surface choppiness can maximize the output voltage V_teng_.

(1) $$V_{teng}=-\frac{q}{A\varepsilon_{0}}\left(\frac{d}{\varepsilon_{r}}+x\left(t\right)\right)+\frac{\sigma x\left(t\right)}{\varepsilon_{r}}$$

Equation (1) uses contact surface area A. A = L × W, where q denotes the transferred charges between electrodes. The air permittivity (ε_0_) and relative permittivity of tribo-material (ε_r_) are represented. Here, V_teng_ was dominantly varied by x(t) when x(t) and d were changed with 0 to 20 mm and 0 to 300 μm, respectively. Additionally, Figure 6 shows the variation of the V_teng_ when d is 0 to 300 μm and x(t) is 0 to 20 mm, but it rarely changes by d. This is due to the non-uniform electric field of connected triboelectric surfaces, which supports the theoretical study. The more optimal results are achieved with a 6 mm air gap for the hybrid NG. Figure 6 shows spin coater-created depths of UV-curable PU tested in hybrid mode to determine triboelectric layer thickness.

**Figure 7:**
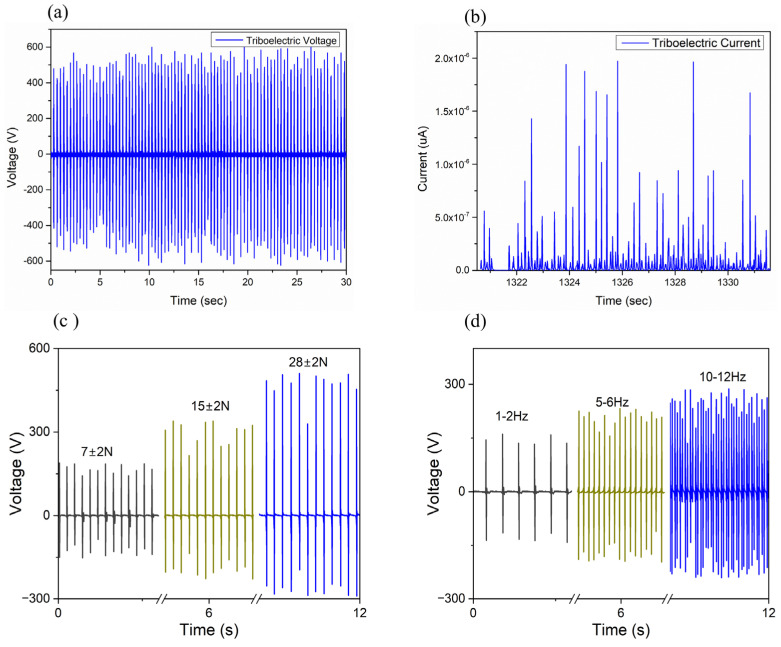
(**a**,**b**) TENG Voltage (V) and Current (µA) graphs with respect to time (s). (**c**) Voltage output response with varying applied forces. (**d**) Frequency-dependent output response of the TENG device.

With the help of a finger-tapping mechanism, the produced TENG receives its input stress. For electrical characterization, a full-bridge rectifier was employed alongside a voltage divider circuit, and the rectified open-circuit voltage (V_OC_) peaks were measured using an oscilloscope. The maximum V_OC_ peak recorded was approximately 500 V, generated in response to a 30 N force applied at a frequency of 3–5 Hz. As the load resistance increased, the voltage across the load also increased, demonstrating the device’s ability to handle high-resistance loads effectively. The maximum power density was observed at a load resistance of 10 MΩ, likely due to impedance matching with the oscilloscope. These results highlight the importance of load impedance optimization for achieving peak power output. The output voltage was measured using an Instek GDS-810C 100 MHz Digital Storage Oscilloscope (Qingdao Hantek Electronic Co., Ltd., USA). The TENG produces a maximum output voltage of approximately 500 V and creates a current of 2 μA, as seen in Figure 7a,b. Because they depend on capacitive modes and air gaps for resistance, TENGs usually have high output impedances. The results show that the proposed TENG has a lot of potential and could be used for many things, such as making energy and sensors that run on their power. The output results of voltages and currents are shown in Figure 7a,b. In Figure 7c, when the TENG operates in vertical contact separation mode, it shows the process of improving the electrical output characteristics. Using different frequencies, experiments were performed to obtain optimal data for the conditions under which the TENG device operates. After determining the optimized structural and geometrical parameters, an enhanced TENG device was fabricated and further analyzed for sensitivity under varying forces and frequencies. As illustrated in Figure 7c, the device generated approximately 184 V when subjected to a force of 7 ± 2 N. With an increase in applied force, the output voltage also increased, reaching peaks of over 550 V at a force of 28 ± 2 N. Additionally, the effect of frequency on the device performance was evaluated while maintaining a constant force of 15 ± 2 N. A significant increase in output voltage was observed with increasing frequency, up to 12 Hz in Figure 7d. Beyond this frequency, however, no substantial improvement in output performance was noted, indicating a saturation point in the device’s frequency response.

**Figure 8:**
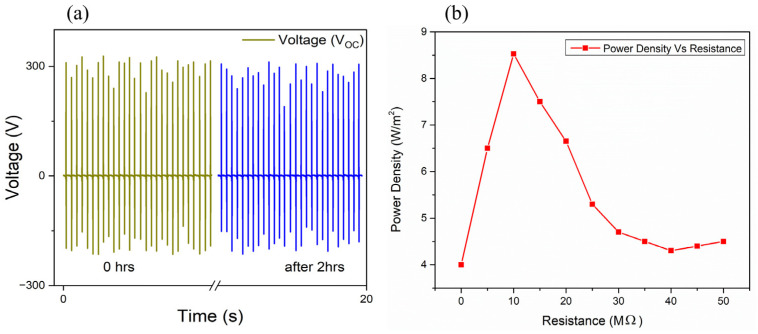
(**a**) Output voltage at 0 h and after 2 h of continuous operation. (**b**) Power Density vs. Resistance.

As the load resistance increased, the voltage across the load also increased, demonstrating the device’s ability to handle high-resistance loads effectively. The maximum power density was observed at a load resistance of 10 MΩ, likely due to impedance matching with the oscilloscope. TENGs’ high voltage and low current characteristics result in mega-ohm internal impedance. Maximum power transfer happens when external load resistance meets the device’s internal impedance. Many studies on polyurethane-based TENGs show that they produce large amounts of power when connected to load resistances between 1 MΩ and 100 MΩ, with 10 MΩ being the best choice. The highest power density at 10 MΩ is obtained by ensuring that the external load matches the internal impedance of the polyurethane-based TENG. Experimental data, usually power–resistance charts, show this in Figure 8b. These results highlight the importance of load impedance optimization for achieving peak power output. To evaluate the stability and durability of the TENG device, continuous force was applied using a force applicator set to approximately 15 N with a frequency of 3–5 Hz. The output voltage was recorded initially and then again after two hours of operation, as shown in Figure 8a.

**Figure 9:**
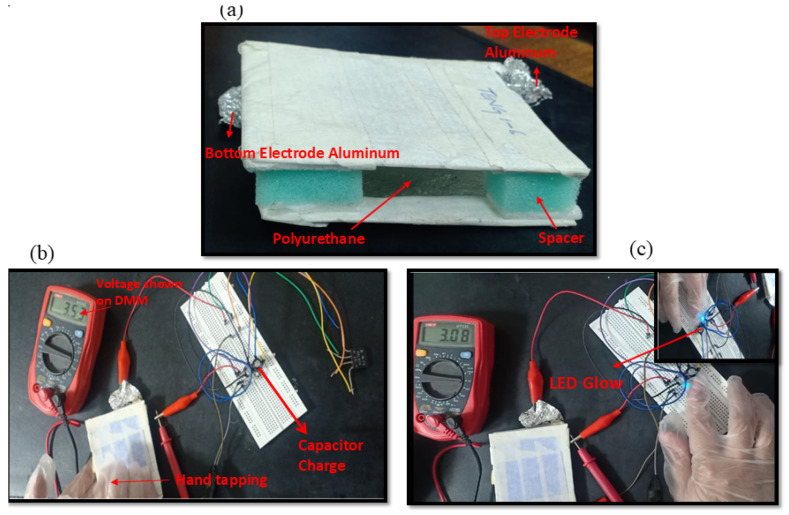
(**a**) Full image of the fabricated TENG. (**b**) Capacitor charging by the TENG device. (**c**) LED’s glow after capacitor charging.

The designed TENG is shown in Figure 9 (full image), with the arrowhead indicating the top and bottom electrodes made of aluminum, the polyurethane, and the sponge spacers. In Figure 9b, the circuit shows the charging of the capacitor with the help of continuous hand tapping on the TENG. DMM shows the voltage that charges the capacitor at 3.53 V to illuminate the LED. In Figure 9c, when the capacitor stores the charges at 3.53 V, the button is used to toggle an LED. When the button is pushed, the LED will glow, as shown in Figure 9c.

## 4. Conclusions

This study project encompasses the design, development, and characterization of a newly created device. In this work, a device is developed that uses UV-curable polyurethane to deliver significant electric power. The assembled devices produced an open-circuit voltage of around 500 V and a current of about 2 µA and showed high performance with a power density of 8.53 W/m^2^. The developed device can serve as an energy-producing solution in wireless communication and monitoring systems for self-sufficient applications, in addition to charging a small battery and capacitor. For real applications, the industrialized TENG device displayed unchanging cyclic charging and discharging properties that are very significant. Moreover, for practical and real-time applications, the TENG device was used to produce mechanical energies with triboelectric effects. It is demonstrated from previous studies that the invented TENG device powerfully converts mechanical activities into electricity.

## Data Availability

Information will be provided upon appropriate request.

## References

[1] Han Y., Zhang Q., Wu L. (2020). Influence on the adsorption of phenol on single-walled carbon nanotubes caused by NaCl and an electrostatic field in saline. Desalination.

[2] Bu C., Li F., Yin K., Pang J., Wang L., Wang K. (2020). Research Progress and Prospect of Triboelectric Nanogenerators as Self-Powered Human Body Sensors. ACS Appl. Electron. Mater..

[3] Shi Q., He T., Lee C. (2019). More than energy harvesting—Combining triboelectric nanogenerator and flexible electronics technology for enabling novel micro-/nano-systems. Nano Energy.

[4] Wang M., Shi X., Liu W., Zou F., Hua P., Zhang M. (2022). A zwitterionic polyurethane-based self-healing triboelectric nanogenerator for efficient self-powered sensing. Mater. Lett..

[5] Jiang Y., Dong K., Li X., An J., Wu D., Peng X., Yi J., Ning C., Cheng R., Yu P. (2020). Stretchable, Washable, and Ultrathin Triboelectric Nanogenerators as Skin-Like Highly Sensitive Self-Powered Haptic Sensors. Adv. Funct. Mater..

[6] Palaniappan V., Bazuin B.J., Atashbar M.Z., Masihi S., Zhang X., Emamian S., Bose A.K., Maddipatla D., Hajian S., Panahi M. A Flexible Triboelectric Nanogenerator Fabricated Using Laser-Assisted Patterning Process. Proceedings of the 2019 IEEE SENSORS Conference.

[7] Gao W.C., Wu W., Chen C.Z., Zhao H., Liu Y., Li Q., Huang C.-X., Hu G.-H., Wang S.-F., Shi D. (2022). Design of a Superhy-drophobic Strain Sensor with a Multilayer Structure for Human Motion Monitoring. Appl. Mater. Interfaces.

[8] De Sousa F.D.B., Scuracchio C.H., Hu G.H., Hoppe S. (2016). Effects of processing parameters on the properties of microwave-devulcanized ground tire rubber/polyethylene dynamically revulcanized blends. Appl. Polym. Sci..

[9] Noon M.A.A., Shah I., Tipu J.A.K., Arif M., Saeed M.U.B., Qzai S.I., Sharif M., Sharif A. (2024). Process and production enhancement through codigestion in biogas generation. Environ. Prog. Sustain. Energy.

[10] Wang Z.L. (2014). Triboelectric nanogenerators as new energy technology and self-powered sensors—Principles, problems and perspectives. Faraday Discuss..

[11] Anwar B., Ali S., Shah I., Aslam Noon M.A., Tipu J.A.K., Arif M. (2023). Proposed optimized energy plan for the largest island of South Korea based on full renewable energy resources. J. Process Mech. Eng..

[12] Zhang X., He D., Palaniappan V., Maddipatla D., Yang Q., Atashbar M.Z. A Novel Graphite/PDMS based Flexible Triboelectric Nanogenerator. Proceedings of the 2021 IEEE International Conference on Flexible and Printable Sensors and Systems (FLEPS).

[13] Huang T., Lu M., Yu H., Zhang Q., Wang H., Zhu M. (2015). Enhanced Power Output of a Triboelectric Nanogenerator Composed of Electrospun Nanofiber Mats Doped with Graphene Oxide. Sci. Rep..

[14] Shao Y., Luo C., Deng B.W., Yin B., Yang M.B. (2020). Flexible porous silicone rubber-nanofiber nanocomposites generated by supercritical carbon dioxide foaming for harvesting mechanical energy. Nano Energy.

[15] Shaukat R.A., Choi J., Jeong C.K. (2023). Eco-Friendly Powder and Particles-Based Triboelectric Energy Harvesters. J. Korean Powder Metall. Inst..

[16] Hassan G., Khan F., Hassan A., Ali S., Bae J., Lee C.H. (2017). A flat-panel-shaped hybrid piezo/triboelectric nanogenerator for ambient energy harvesting. Nanotechnology.

[17] Ali I., Hassan G., Shuja A. (2022). Fabrication of self-healing hybrid nanogenerators based on polyurethane and ZnO for harvesting wind energy. J. Mater. Sci. Mater. Electron..

[18] Sun W., Luo N., Liu Y., Li H., Wang D. (2022). A New Self-Healing Triboelectric Nanogenerator Based on Polyurethane Coating and Its Application for Self-Powered Cathodic Protection. ACS Appl. Mater. Interfaces.

[19] Liu Y., Hu C. (2020). Triboelectric nanogenerators based on elastic electrodes. Nanoscale.

[20] Matsunaga M., Hirotani J., Kishimoto S., Ohno Y. (2020). High-output, transparent, stretchable triboelectric nanogen-erator based on carbon nanotube thin film toward wearable energy harvesters. Nano Energy.

[21] Qin H., Cheng G., Zi Y., Gu G., Zhang B., Shang W., Yang F., Yang J., Du Z., Wang Z.L. (2018). High Energy Storage Efficiency Triboelectric Nanogenerators with Unidirectional Switches and Passive Power Management Circuits. Adv. Funct. Mater..

[22] Yi F., Lin L., Niu S., Yang P.K., Wang Z., Chen J., Zhou Y., Zi Y., Wang J., Liao Q. (2015). Stretchable-Rubber-Based Triboelectric Nano-generator and Its Application as Self-Powered Body Motion Sensors. Adv. Funct. Mater..

[23] Wang W., Yan J., Wang X., Pang H., Sun C., Sun Y., Wang L., Zhang D. (2025). Research on the Performance of a Liquid–Solid Triboelectric Nanogenerator Prototype Based on Multiphase Liquid. Micromachines.

[24] Feng Y., Huang X., Liu S., Guo W., Li Y., Wu H. (2019). A self-powered smart safety belt enabled by triboelectric nan-ogenerators for driving status monitoring. Nano Energy.

[25] Patel T., Kim M.P., Park J., Lee T.H., Nellepalli P., Noh S.M., Jung H.W., Ko H., Oh J.K. (2020). Self-Healable Reprocessable Triboelectric Nanogenerators Fabricated with Vitrimeric Poly(hindered Urea) Networks. ACS Nano.

[26] Ma H.Z., Zhao J.N., Tang R., Shao Y., Ke K., Zhang K., Yin B., Yang M.-B. (2023). Polypyrrole@CNT@PU Conductive Sponge-Based Triboelectric Nanogener-ators for Human Motion Monitoring and Self-Powered Ammonia Sensing. ACS Appl. Mater. Ad. Interfaces.

[27] Shateri A.A.A., Zhuo F., Shuaibu N.S., Wan R., Xu L., Hazarika D., Gyawawli B., Wang X. (2025). Generating a Full Cycle of Alternative Current Using a Triboelectric Nanogenerator for Energy Harvesting. Micromachines.

[28] Sun J., Pu X., Liu M., Yu A., Du C., Zhai J., Zhai J., Hu W., Wang Z.L. (2018). Self-Healable, Stretchable, Transparent Triboelectric Nanogenerators as Soft Power Sources. ACS Nano.

[29] Lee J.H., Hinchet R., Kim S.K., Kim S., Kim S.W. (2015). Shape memory polymer-based self-healing triboe-lectric nanogenerator. Energy Environ. Sci..

[30] Parida K., Thangavel G., Cai G., Zhou X., Park S., Xiong J., Lee P.S. (2019). Extremely stretchable and self-healing conductor based on thermoplastic elastomer for all-three-dimensional printed triboelectric nanogenerator. Nat. Commun..

[31] Doganay D., Cicek M.O., Durukan M.B., Altuntas B., Agbahca E., Coskun S., Unalan H.E. (2021). Fabric based wearable triboelectric nanogenerators for human machine interface. Nano Energy.

[32] Zhu P., Mu S., Huang W., Sun Z., Lin Y., Chen K., Pan Z., Haghighi M.G., Sedghi R., Wang J. (2024). Soft mul-tifunctional neurological electronic skin through intrinsically stretchable synaptic transistor. Nano Res..

[33] Srither S.R., Shankar Rao D.S., Krishna Prasad S. (2018). Triboelectric Nanogenerator Based on Biocompatible and Easily Available Polymer Films. Chem. Sel..

[34] Jiang J., Guan Q., Liu Y., Sun X., Wen Z. (2021). Abrasion and Fracture Self-Healable Triboelectric Nanogenerator with Ultrahigh Stretchability and Long-Term Durability. Adv. Funct. Mater..

[35] Park S.I., Lee D.M., Kang C.W., Lee S.M., Kim H.J., Ko Y.-J., Kim S.-W., Son S.U. (2021). Triboelectric energy harvesting using conjugated microporous polymer nanoparticles in polyurethane films. Mater. Chem. A.

[36] Li C., Yin Y., Wang B., Zhou T., Wang J., Luo J., Tang W., Cao R., Yuan Z., Li N. (2017). Self-Powered Electrospinning System Driven by a Triboelectric Nanogenerator. ACS Nano.

[37] Dharmasena R.D.I.G., Wijayantha K.G.U. (2021). Theoretical and experimental investigation into the asymmetric external charging of Triboelectric Nanogenerators. Nano Energy.

[38] Yang S., Larionova T., Kobykhno I., Klinkov V., Shalnova S., Tolochko O. (2024). Graphene-Doped Thermoplastic Polyu-rethane Nanocomposite Film-Based Triboelectric Nanogenerator for Self-Powered Sport Sensor. Nanomaterials.

[39] Flores J.M.B., Rivera J.M., Ortiz G.R., Ahuactzi I.F.H., Chavarria J.J.C., Melecio H.A.A., Sanchez P.D.A., Ceron V.H.A. (2024). Energy harvesting through the triboelectric nanogenerator (TENG) based on polyurethane/cellulose nanocrystal. Int. J. Renew. Energy Dev..

